# Appendicular Skeletal Muscle Index and HbA1c Evaluate Liver Steatosis in Patients With Metabolic Associated Fatty Liver Disease

**DOI:** 10.3389/fmed.2022.919502

**Published:** 2022-07-06

**Authors:** Rui Jin, Xiaoxiao Wang, Xiaohe Li, Jia Yang, Baiyi Liu, Lai Wei, Feng Liu, Huiying Rao

**Affiliations:** ^1^Peking University People’s Hospital, Peking University Hepatology Institute, Beijing Key Laboratory of Hepatitis C and Immunotherapy for Liver Diseases, Beijing International Cooperation Base for Science and Technology on NAFLD Diagnosis, Beijing, China; ^2^Beijing Tsinghua Changgung Hospital, Tsinghua University, Beijing, China

**Keywords:** appendicular skeletal muscle index (ASMI), HbA1c, liver, steatosis, metabolic associated fatty liver disease (MAFLD)

## Abstract

**Background and Aim(s):**

Liver steatosis, as the main feature of metabolic associated fatty liver disease (MAFLD), was associated with the progression of liver fibrosis and metabolic syndrome, which needed to be estimated accurately. In this study, we explored the significance of appendicular skeletal muscle index (ASMI) in evaluating liver steatosis of MAFLD patients.

**Methods:**

Eight hundred and ninety-nine cases with MAFLD from 2017 to 2018 National Health and Nutrition Examination Surveys (NHANES) database were included. All the analyzed data were obtained from NHANES database. The association between ASMI and liver steatosis were evaluated using R and EmpowerStats.

**Results:**

MAFLD individuals were randomly divided into a training (*n* = 450) and validation cohort (*n* = 449). In univariate analysis, HbA1c, arms fat, arms lean mass, legs lean mass, trunk lean mass, total fat, total lean mass and ASMI were significantly associated with liver steatosis (*p* < 0.05). Multivariate analysis showed that HbA1c (OR: 1.6732; 95% CI: 1.2753–2.1929, *p* = 0.0002) and ASMI (OR: 1.6723; 95% CI: 1.1760–2.5204, *p* = 0.0052) were independently associated with severe liver steatosis. ASMI accurately evaluated severe liver steatosis with an AUROC of 0.73 and 0.81 in training and validation cohort, respectively. Compared with ASMI only, ASMI combined with HbA1c improved the AUROC to 0.85 and 0.88. Furthermore, the AUROC of our model was superior to FLI in the evaluation of liver steatosis.

**Conclusion:**

ASMI combined with HbA1c has good evaluation value for liver steatosis in MAFLD patients, which might be beneficial for the management of MAFLD clinically.

## Introduction

Non-alcoholic fatty liver disease (NAFLD) was the most common liver disease globally, affecting about a quarter of the population. It imposed a significant health and economic burden on all societies (1). Most studies confirmed that the prevalence of NAFLD was often accompanied by the occurrence of a variety of metabolic disorders, which might aggravate liver injury of NAFLD (2, 3). Due to the heterogeneity of patients with NAFLD, a panel of experts put forward that the metabolic (dysfunction) associated fatty liver disease (MAFLD) might be a more appropriate overarching term (4). The most significant advantages of diagnostic criteria in MAFLD was the definition of liver steatosis and the presence of metabolic abnormalities (5). It had been reported that significant steatosis was associated with fibrosis progression in patients with NAFLD, and the degree of liver steatosis was associated with metabolic syndrome and cardiovascular risk (3). Therefore, it was essential to estimate liver steatosis accurately in patients with NAFLD/MAFLD. At present, liver biopsy was still the gold standard for measuring liver fat content, however, it was an invasive operation, making it unreasonable to evaluate liver steatosis routinely. Therefore, it was necessary for us to find a simple and effective non-invasive method to accurately evaluate liver steatosis in MAFLD patients.

Appendicular skeletal muscle index (ASMI) was a parameter that reflects the weight of appendicular muscles per square meter of height (kg/m^2^) (6). Usually, ASMI was often used to evaluate skeletal muscle in patients with chronic liver disease (e.g., cirrhosis and end-stage liver disease), so as to reflect the body condition of patients indirectly (6, 7). ASMI also was associated with the progression of chronic metabolic diseases. For instance, the skeletal muscle mass index was negatively correlated with liver steatosis in males with type 2 diabetes (8). Furthermore, researchers found that the destruction of the relationship between skeletal muscle and liver accelerated the progression of NAFLD (9, 10). The underlying mechanism was that excessive energy produced by skeletal muscle during exercise might be stored in the liver in the form of lipids, which would increase the accumulation of liver fat (11). However, the correlation between ASMI and the degree of liver steatosis was unclear. In this study, we will estimate the value of ASMI in evaluating liver steatosis in patients with MAFLD through a population-based data from National Health and Nutrition Examination Surveys (NHANES).

## Materials and Methods

### Study Design and Participants

Datasets from 2017 to 2018 NHANES required for the cross-sectional study were downloaded from the NHANES web site.^1^ The NHANES database was a nationally representative survey of the United States conducted annually by CDC’s National Center for Health Statistics (CDC/NCHS), often used in NAFLD (MAFLD) research (12). The study was approved by the NCHS research ethics review board. Informed consents were obtained from all participants in this study. The study protocol also conformed to the ethical guidelines of the Declaration of Helsinki revised in 2013.

**FIGURE 1:**
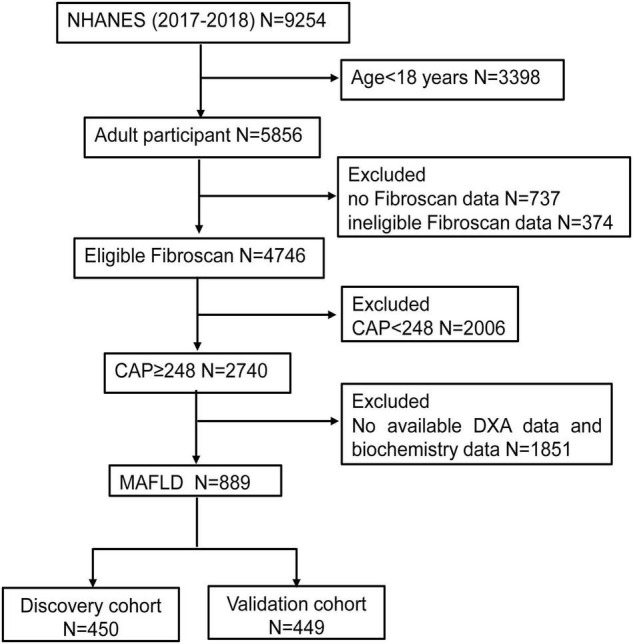
The flow chart of participants selection. NHANES, National Health and Nutrition Examination Surveys; CAP, Controlled attenuation parameter; DXA, dual-energy X-ray absorptiometry; MAFLD, Metabolic associated fatty liver disease.

In total, 9,254 individuals were initially identified from the database 2017–2018 NHANES. Individuals who were younger than 18 years (*n* = 3,398), without Fibroscan data (*n* = 737) or with ineligible Fibroscan data (*n* = 374), CAP < 248 (*n* = 2,006) and without dual-energy X-ray absorptiometry (DXA) data and biochemistry data (*n* = 1,851) were excluded. As a consequence, 889 individuals were included in the final analysis and divided into discovery cohort (*n* = 450) and validation cohort (*n* = 449) randomly (Figure 1).

### Diagnostic Criteria of Metabolic Associated Fatty Liver Disease

Metabolic associated fatty liver disease (MAFLD) was defined by the evidence of liver steatosis in adults (CAP ≥ 248 detected by VCTE in this study) (13, 14) and one of the following three criteria, namely overweight/obesity (body mass index, BMI ≥ 25 kg/m^2^), T2DM or evidence of metabolic dysregulation. Furthermore, among lean/normal weight individuals (BMI < 25 kg/m^2^) with liver steatosis who did not have T2DM, the presence of metabolic dysregulation was defined as the presence of two or more of the following metabolic risk abnormalities: (1) waist circumference ≥ 102 cm in men or 88 cm in women, (2) blood pressure (BP) ≥ 130/85 mmHg, (3) serum triglycerides (TG) ≥ 1.70 mmol/L, (4) high-density lipoprotein cholesterol (HDL-C) < 1.0 mmol/L for men or < 1.3 mmol/L for women, (5) prediabetes [i.e., fasting plasma glucose (FPG) 5.6–6.9 mmol/L, or 2-h post-load glucose levels (2 h PBG) 7.8–11.0 mmol/L or HbA1c 5.7–6.4%], (6) HOMA-IR score ≥ 2.5, and (7) plasma C-reactive protein (CRP) level > 2 mg/L (5).

### Vibration-Controlled Transient Elastography

Vibration-controlled transient elastography (VCTE) was performed using the Fibroscan model 502 V2 Touch (Echosens, Paris, France) equipped with both a medium (M) or extra-large (XL) probes. Examinations were considered reliable only if at least 10 liver stiffness measurements (LSM) were obtained after a fasting time of at least 3 h, with an interquartile range/median <30%. In this study, the severity of liver steatosis in MAFLD patients was defined by CAP value detected by VCTE [Mild/Moderate steatosis (S1-S2: 248–279 dB/m) and Severe steatosis (S3 ≥ 280 dB/m)] (13, 15). Mild/Moderate steatosis defined as non-Severe steatosis.

### Body Composition Measurement

Body composition such as lean mass, fat mass, and bone mineral density in MAFLD patients was assessed by DXA scanning. Scans were acquired on Hologic QDR-4500A fan-beam densitometers (Hologic, Inc., Bedford, Massachusetts) using software version Apex 3.2. Further details of the DXA examination were documented on the NHANES website (see text footnote 1). Skeletal muscle indices were calculated by dividing lean mass of the respective body compartments with height squared: appendicular skeletal muscle index (ASMI) = (arms lean mass + legs lean mass)/height^2^, arm muscle index (AI) = arms lean mass/height^2^, leg muscle index (LI) = legs lean mass/height^2^.

### Variables

ASMI, as the independent variable, was measured by DXA scanning during the mobile examination center (MEC) visit and calculated by researchers, and CAP, as dependent variables were determined by VCTE measurement. For covariables, continuous variables included age (years), anthropometric measures, FPG (mmol/L), total cholesterol (TC) (mmol/L), TG (mmol/L), HDL-C (mmol/L), total bilirubin (TBIL, μmol/L), alanine aminotransferase (ALT, U/L), aspartate aminotransferase (AST, U/L), γ-glutamyl transpeptidase (GGT, U/L), albumin (ALB, g/L), alkaline phosphatase (ALP, U/L), uric acid (UA, μmol/L), HbA1c (%); categorical variables included gender, race, metabolic diseases, and average alcoholic drinks.

### Statistical Analysis

We constructed univariate and multivariate analysis to explore the association between ASMI and severity of steatosis in MAFLD patients. Furthermore, by logistic regression analysis, we established calculated the AUROC of different models for severe liver steatosis, the dependent variable of the study was dichotomous variable (non-Severe steatosis or Severe steatosis). All statistical analyses were conducted by using R 4.0.2^2^ and EmpowerStats.^3^ Sample weights were used to calculate all estimates according to the analytical guideline provided by NCHS. A *p*-value < 0.05 was considered as statistically significant.

## Results

### Baseline and Dual-Energy X-ray Absorptiometry Characteristics of Study Population

A total of 899 individuals with MAFLD were extracted from 2017 to 2018 NHANES database according to the screening criteria in this study. Subsequently, the cohort was randomly divided into a training cohort (*n* = 450) and validation cohort (*n* = 449) (Figure 1). The mean age in the training cohort was 41.2 years and 255 individuals (56.7%) were male, while the mean age in the validation cohort was 41.3 years and 261 individuals (58.1%) were male. No matter in training cohort or validation cohort, MAFLD patients showed high BMI (>35 kg/m^2^), waist circumference (>104 cm) and hip circumference (>109 cm). In our research population, MAFLD patients often have a variety of metabolic diseases (Hypertension in training/validation cohort: 13.3%/15.8%; Diabetes in training/validation cohort: 9.1%/10.3%) and a history of heavy drinking (Table 1). Except for PLT (*p* = 0.007), LSM data (*p* = 0.048) and the prevalence of thyroid problem (*p* = 0.026), no statistically differences were observed in anthropometrics data, blood test parameters, CAP data, prevalence of metabolic disorders (*p* > 0.05). The details of the baseline characteristics of MAFLD patients in two cohorts were presented in Table 1. We also collected DXA data, including lean mass, fat mass and Bone mineral content (BMC) in total body, arms, legs and trunk, and DXA index, including AI, LI, and ASMI. We observed that there were no differences in the DXA characteristics between the training and validation cohorts (*p* > 0.05) (Table 2).

**TABLE 1:** Baseline characteristics of MAFLD patients in the 2017–2018 NHANES.

Variables	Training Cohort (*n* = 450)	Validation Cohort (*n* = 449)	*P*-value
Age (year)	41.2 ± 11.4	41.3 ± 12.2	0.964
Gender, n (%)			0.683
Male	255 (56.7)	261 (58.1)	
Female	195 (43.3)	188 (41.9)	
**Anthropometrics**			
Weight (kg)	90.8 ± 18.0	89.4 ± 18.2	0.240
BMI (kg/m^2^)	31.9 ± 5.4	31.5 ± 6.4	0.249
Waist circumference (cm)	105.7 ± 12.6	104.5 ± 13.5	0.155
Hip circumference (cm)	110.0 ± 10.9	109.6 ± 12.3	0.527
Race, n (%)			0.626
Mexican American	68 (15.2)	76 (16.9)	
Other Hispanic	37 (8.3)	44 (9.7)	
Non-Hispanic	320 (71.2)	302 (67.2)	
Other races	24 (5.3)	28 (6.2)	
**Blood test**			
FPG (mmol/L)	5.6 ± 1.8	5.6 ± 2.0	0.974
HbA1c (%)	5.7 ± 1.0	5.7 ± 1.0	0.244
ALT (U/L)	28.9 ± 18.8	27.6 ± 18.6	0.323
AST (U/L)	23.5 ± 13.5	22.9 ± 11.4	0.472
ALP (U/L)	78.3 ± 23.8	78.2 ± 19.3	0.915
GGT (U/L)	35.0 ± 41.0	34.8 ± 30.8	0.965
ALB (g/L)	41.1 ± 3.2	41.5 ± 3.0	0.078
TBIL (μmol/L)	7.4 ± 4.9	7.6 ± 3.9	0.456
TG (mmol/L)	2.0 ± 1.5	2.0 ± 1.550	0.694
TC (mmol/L)	5.1 ± 1.0	5.0 ± 1.0	0.487
HDL-C (mmol/L)	1.3 ± 0.3	1.2 ± 0.4	0.397
Creatinine (μmol/L)	77.7 ± 49.0	75.2 ± 17.6	0.302
UA (μmol/L)	331.3 ± 77.2	340.7 ± 82.9	0.080
PLT (10^9^/L)	257.0 ± 59.6	246.0 ± 62.5	0.007
HGB (g/dL)	14.5 ± 1.5	14.5 ± 1.4	0.673
**VCTE**			
CAP (dB/m), n (%)			0.772
S1 (248–267)	76 (17.2)	76 (16.9)	
S2 (268–279)	68 (15.1)	61 (13.5)	
S3 (≥280)	305 (67.7)	313 (69.6)	
IQRc			0.225
	35.4 ± 18.7	33.9 ± 17.7	
LSM (kPa), n (%)			0.048
F0 (<6.3)	78 (17.2)	76 (16.9)	
F1 (6.3–8.2)	68 (15.1)	61 (13.5)	
F2 (8.3–10.4)	20 (4.6)	15 (3.3)	
F3 (10.5–12.4)	3 (0.7)	8 (1.7)	
F4 (≥12.5)	6 (1.3)	11 (2.2)	
IQRe			0.659
	0.8 ± 0.6	0.8 ± 0.7	
Ratio: stiffness IQRe/median E			0.758
	13.8 ± 6.1	13.6 ± 6.0	
Hypertension	60 (13.3)	71 (15.8)	0.299
Diabetes	41 (9.1)	46 (10.3)	0.644
Gout	12 (2.7)	14 (3.1)	0.299
CHD	5 (1.0)	3 (0.7)	0.287
Thyroid problem	38 (8.4)	21 (4.6)	0.026
**Average alcoholic drinks, n (%)**			
<4 drinks/day	244 (54.3)	283 (63.0)	
4–8 drinks/day	60 (13.3)	29 (6.4)	
>8 drinks/day	8 (1.7)	13 (2.9)	
NA	138 (30.7)	124 (27.6)	

*Continuous variables are shown as mean ± standard deviation (SD). Categorical values are shown as n (%). MAFLD, Metabolic-associated fatty liver disease; BMI: body mass index; FPG, fasting plasma glucose; HbA1c, ALT, alanine aminotransferase; AST, aspartate aminotransferase; ALP, alkaline phosphatase; GGT, γ-glutamyl transpeptidase; ALB, albumin; TBIL, total bilirubin; TG, triglycerides; TC, Total cholesterol; HDL-C, high-density lipoprotein cholesterol; UA, uric acid; PLT, platelet; HGB, hemoglobin; VCTE, Vibration Controlled Transient Elastography; CAP, controlled attenuation parameter; IQRc, CAP interquartile range; LSM, liver stiffness measurements; IQRe, Stiffness E interquartile range; CHD, Coronary Heart Disease. NA, Not available.*

**TABLE 2:** DXA characteristics of MAFLD patients in the 2017–2018 NHANES.

Variables	Training cohort (*n* = 450)	Validation Cohort (*n* = 449)	*P*-value
**Absolute value**			
Total lean mass (kg)	56.7 ± 12.5	55.8 ± 11.6	0.264
Total fat mass (kg)	32.0 ± 95.4	31.4 ± 10.7	0.323
Total BMC (g)	2443.6 ± 454.7	2436.7 ± 409.3	0.813
Arms lean mass (kg)	6.9 ± 2.1	6.7 ± 2.0	0.127
Arms fat (kg)	4.0 ± 1.5	3.8 ± 1.5	0.043
Arms BMC (g)	390.3 ± 97.6	384.0 ± 85.4	0.304
Legs lean mass (kg)	18.0 ± 4.3	17.9 ± 4.1	0.589
Legs fat (kg)	10.5 ± 3.8	10.5 ± 4.2	0.966
Legs BMC (g)	908.1 ± 204.0	915.7 ± 184.4	0.559
Trunk lean mass (kg)	28.6 ± 6.2	28.0 ± 5.6	0.159
Trunk fat (kg)	15.6 ± 5.3	15.8 ± 5.6	0.657
Trunk BMC (g)	632.1 ± 130.2	629.6 ± 121.4	0.773
**Index**			
Arms muscle index (AI) (kg/m^2^)	2.39 ± 0.56	2.32 ± 0.57	0.061
Legs muscle index (LI) (kg/m^2^)	6.28 ± 1.08	6.23 ± 1.18	0.570
ASMI (kg/m^2^)	8.66 ± 1.56	8.55 ± 1.67	0.291

*MAFLD, Metabolic-associated fatty liver disease; BMC, Bone mineral content; AI, Arms muscle index; LI, Legs muscle index; ASMI, Appendicular skeletal muscle index.*

### Model Development

Univariate and multivariate logistic regression analysis were used to analyze the related factors for liver steatosis in MAFLD patients. According to the univariate analysis, HbA1c, arms fat, arms lean mass, legs lean mass, trunk lean mass, total fat, total lean mass, and ASMI were significantly associated with liver steatosis (*p* < 0.05). These significant variables were further conducted to the multivariate analysis. Multivariate analysis showed that HbA1c [odds ratio (OR): 1.6732; 95% CI: 1.2753–2.1929, *p* = 0.0002] and ASMI (OR, 1.6723; 95% CI: 1.1760–2.5204, *p* = 0.0052) were independently associated with severe liver steatosis in MAFLD patients (Table 3).

**TABLE 3:** Univariate and multivariate analysis of variables associated with liver steatosis.

	Univariate analysis		Multivariate analysis	
Variables	OR (95%CI)	*P*-value	OR (95%CI)	*P*-value
Age (year)	0.9980 (0.9494, 1.0490)	0.9363		
Gender, n (%)	0.4040 (0.1079, 1.5127)	0.1785		
Waist circumference (cm)	1.0702 (1.0296, 1.1125)	0.0006		
Hip circumference (cm)	1.0366 (0.9940, 1.0812)	0.0934		
BMI (kg/m^2^)	1.1265 (1.0400, 1.2201)	0.0035		
ALT (U/L)	1.0266 (1.0096, 1.0439)	0.0020		
AST (U/L)	1.0310 (1.0101, 1.0523)	0.0035		
GGT (U/L)	1.0023 (0.9939, 1.0108)	0.5912		
ALB (g/L)	0.9981 (0.8351, 1.1930)	0.9835		
ALP (U/L)	1.0050 (0.9821, 1.0285)	0.6710		
CRE (μmol/L)	0.9974 (0.9730, 1.0224)	0.8369		
HDL-C (mmol/L)	0.0981 (0.0091, 1.0523)	0.0551		
HbA1c (%)	1.5812 (1.2275, 2.0370)	0.0004	1.6723 (1.2753, 2.1929)	0.0002
Arms fat (kg)	1.0003 (1.0000, 1.0006)	0.0425		
Arms BMC (g)	1.0031 (0.9974, 1.0089)	0.2850		
Arms lean mass (kg)	1.0003 (1.0000, 1.0006)	0.0336		
Legs fat (kg)	1.0001 (1.0000, 1.0002)	0.2255		
Legs BMC (g)	1.0006 (0.9977, 1.0034)	0.6991		
Legs lean mass (kg)	1.0001 (1.0000, 1.0003)	0.0486		
Trunk fat (kg)	1.0000 (0.9999, 1.0001)	0.8382		
Trunk BMC (g)	1.0027 (0.9982, 1.0073)	0.2366		
Trunk lean mass (kg)	1.0002 (1.0001, 1.0003)	0.0020		
Total fat (kg)	1.0000 (1.0000 1.0001)	0.0389		
Total BMC (g)	1.0027 (0.9982, 1.0073)	0.2366		
Total lean mass (kg)	1.0001 (1.0000, 1.0001)	0.0085		
ASMI (kg/m^2^)	1.6308 (1.1329, 2.3475)	0.0085	1.7216 (1.1760 2.5204)	0.0052

*BMI, body mass index; ALT, alanine aminotransferase; AST, aspartate aminotransferase; GGT, γ-glutamyl transpeptidase; ALB, albumin; ALP, alkaline phosphatase; HDL-C, high-density lipoprotein cholesterol; CRE, creatinine; BMC, Bone mineral content; ASMI, Appendicular skeletal muscle index.*

### Performance of Arms Muscle Index, Legs Muscle Index, Appendicular Skeletal Muscle Index, and Appendicular Skeletal Muscle Index Combined With HbA1c for Evaluating Severe Liver Steatosis in Metabolic Associated Fatty Liver Disease Patients

**FIGURE 2:**
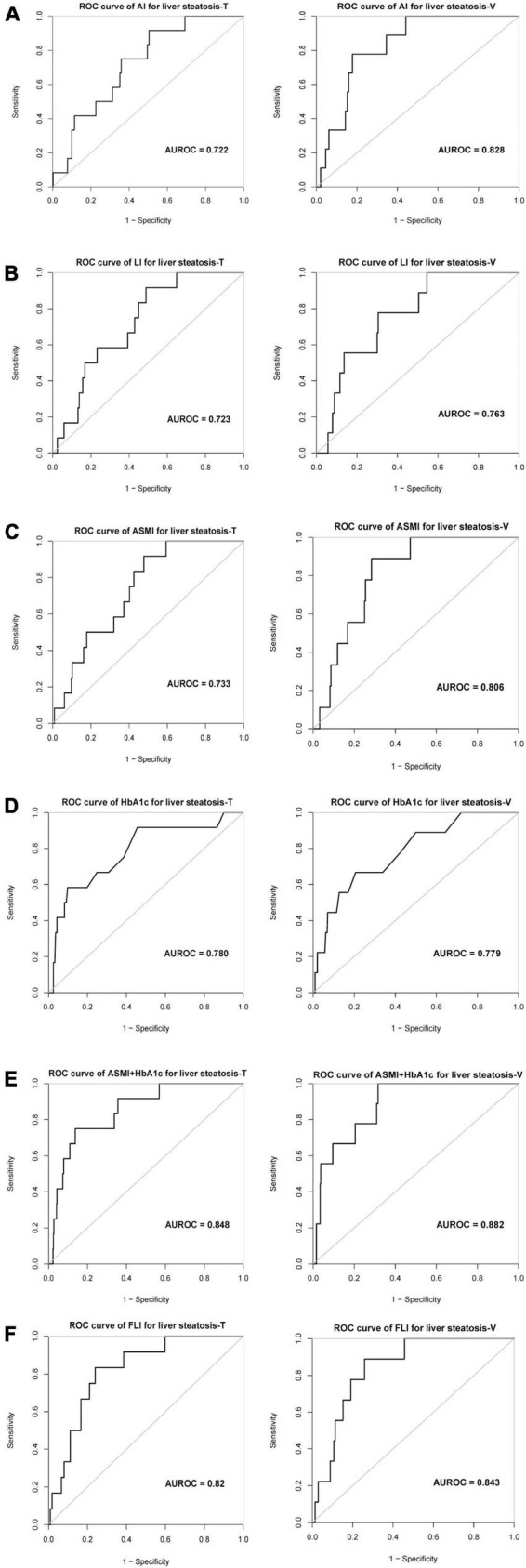
The evaluation value of different indexes on severe liver steatosis of MAFLD patients. ROC curve and AUROC value of **(A)** AI, **(B)** LI, **(C)** ASMI, **(D)** HbA1c, **(E)** ASMI + HbA1c, and **(F)** FLI for liver steatosis in training and validation cohort. AI, Arms muscle index; LI, Legs muscle index; ASMI, Appendicular skeletal muscle index; FLI, Fatty liver index; AUROC, the area under the receiver operating characteristic.

AI accurately evaluated severe liver steatosis in MAFLD patients with an AUROC of 0.72 in training cohort and 0.83 in validation cohort, sensitivities of 92 and 78% and specificities of 50 and 82% were obtained in training cohort and validation cohort, respectively (Table 4 and Figure 2A). The LI evaluated the severity liver steatosis in MAFLD patients with an AUROC of 0.72 in training cohort and 0.76 in validation cohort, sensitivities of 92 and 78% and specificities of 51 and 70% were obtained in training cohort and validation cohort, respectively (Table 4 and Figure 2B). The ASMI demonstrated an AUROC of 0.73 in training cohort and 0.81 in validation cohort for evaluating severe liver steatosis in MAFLD patients. The combination of ASMI and HbA1c improved performance of ASMI only, increasing the AUROC to 0.85 and 0.88, respectively. In training cohort, sensitivity was 92% and specificity was 52% for ASMI alone, turned to 75 and 86% by the addition of HbA1c, respectively. Similarly, in validation cohort, sensitivity was 89% and specificity was 72% for ASMI alone, turned to 100 and 68% by the addition of HbA1c, respectively (Table 4 and Figures 2C–E). Furthermore, using linear regression, the combination of ASMI and HbA1c also could evaluate liver steatosis conveniently and effectively [Liver steatosis (CAP value) = 200.95812 + 6.88228 × ASMI + 7.75809 × HbA1c]. We also calculated the diagnostic value of the fatty liver index (FLI, a non-invasive approach to discriminate individuals with NAFLD) for liver steatosis severity in this study. In training cohort, the FLI had an AUROC of 0.82 for identifying severe liver steatosis in MAFLD patients. Sensitivity and specificity were 83 and 76%, respectively. While in validation cohort, the AUROC was 0.84, the sensitivity was 89% and the specificity was 74% (Table 4 and Figure 2F). The full performance characteristics of AI, LI, ASMI, ASMI combined with HbA1c, as well as FLI were provided in Table 4.

**TABLE 4:** Association of ASMI with severity of steatosis in MAFLD patients.

Models	Cohort	AUROC	Cut-off value	Sensitivity	Specificity
Arm muscle index (AI)	Training	0.72	0.42	0.92	0.50
	Validation	0.83	0.60	0.78	0.82
Leg muscle index (LI)	Training	0.72	0.43	0.92	0.51
	Validation	0.76	0.48	0.78	0.70
ASMI	Training	0.73	0.61	0.92	0.52
	Validation	0.81	0.48	0.89	0.72
HbA1c	Training	0.78	0.46	0.58	0.90
	Validation	0.78	0.61	0.67	0.79
ASMI + HbA1c	Training	0.85	0.61	0.75	0.86
	Validation	0.88	0.68	1.00	0.68
Fatty liver index (FLI)	Training	0.82	0.59	0.83	0.76
	Validation	0.84	0.63	0.89	0.74

*AI, Arms muscle index; LI, Legs muscle index; ASMI, Appendicular skeletal muscle index; FLI, Fatty liver index.*

## Discussion

This was the first and large population-based cross-sectional study that evaluated severe liver steatosis by skeletal muscle index and metabolic disorders in MAFLD patients. In this study, we found that ASMI and HbA1c were associated with liver steatosis in MAFLD patients independently. Notably, ASMI combined with HbA1c achieved a good predictive value for evaluating liver steatosis in MAFLD patients.

Steatosis was a crucial pathological manifestation in the liver of patients with MAFLD (16). It was well-known that advanced steatosis was associated with progression of fibrosis in NAFLD patients (17). Besides, the degree of liver steatosis was related to the occurrence of metabolic syndrome and cardiovascular risk (18). Therefore, an accurate estimation of the liver steatosis was necessary for patients with MAFLD. Previous studies had shown that CAP value detected by VCTE was significantly correlated with the severity of steatosis evaluated by liver biopsy (19), and demonstrated the accuracy of CAP for diagnosis of NAFLD (20–22). Furthermore, Yu et al. demonstrated that CAP detected by VCTE were highly accurate for assessing liver steatosis in patients of MAFLD and T2DM (23). Therefore, CAP value could accurately reflect liver steatosis in MAFLD patients.

In our study, we found that ASMI combined with HbA1c had good diagnostic performance for liver steatosis detected by VCTE in patients with MAFLD. Skeletal muscle had been considered as a vital organ for whole body metabolism, since it was a primary site for glucose uptake and storage, and it was also a reservoir of amino acids stored as protein (24). Previous studies showed that the crosstalk between muscle and liver might play an important role in the pathogenesis of NASH. For instance, reduced appendicular skeletal muscle mass as well as increased visceral fat mass might adversely affect the risk of NAFLD development and progression (25, 26). Many other researches also assumed that low ASM mass or ASMI was associated with more severe liver steatosis and/or hepatocellular ballooning in NAFLD patients (27–29). However, there were no published paper explored the relationship between ASMI and liver steatosis in patients with MAFLD. Intriguingly, our study found ASMI was positively related to liver steatosis in MAFLD patients, which was different from the results in NAFLD patients. Firstly, skeletal muscle was an important organ for storing and releasing lipids in response to fatty acid oversupply, excessive fatty acids and energy storage may lead to the increase the mass of skeletal muscle. In this situation, fatty acids released from skeletal muscle might aggravate insulin resistance and liver steatosis in MAFLD patients (30, 31). Secondly, liver steatosis and ASMI both were affected by BMI (a parameter calculated by height and weight) (32, 33), in our study, most individuals with large ASMI also showed higher height and weight, and the corresponding liver steatosis was more serious. Thirdly, MAFLD was associated with a variety of metabolic abnormalities, which might affect the relationship between ASMI and steatosis. For instance, HbA1c was a metabolic index which was positively associated with liver steatosis in our study. One recent study showed that higher mean HbA1c was associated with higher grade of steatosis and ballooned hepatocytes (34). Indeed, compared with ASMI only, ASMI combined with HbA1c increased the AUROC from 0.73 to 0.85 for assessing severe liver steatosis in MAFLD patients. One previous study reported that FLI was one of the most accurate algorithms for the non-invasive diagnosis of NAFLD in both lean and overweight/obese population (35, 36), while the AUROC of our model was superior to FLI in MAFLD patients. All variables in the non-invasive clinical model established in this study were objective results and easy to obtain, which could be a better evaluation method for liver steatosis in patients with MAFLD.

There were some limitations in this study. Firstly, liver steatosis of MAFLD patients was detected by Fibroscan, not by liver biopsy, because it was impracticable to perform invasive tests such as biopsies in a large population-based study. Secondly, ASMI was a parameter calculated based on the appendicular skeletal muscle mass obtained from dual-energy X-ray absorptiometry (DXA), some medical institutions might not have the objective conditions for this operation. Thirdly, ASMI only assessed muscle weight, muscle length and muscle fiber type were not considered. Finally, there was still a lack of another group of MAFLD patients for external validation. We will perform the validation analysis deeply in the future.

## Conclusion

We constructed a clinical diagnostic method for non-invasive evaluation the degree of steatosis in MAFLD patients, with fewer parameters (ASMI and HbA1c) and high accuracy. The clinical method might be beneficial to reduce the financial cost of liver biopsy and convenient for clinicians to identify the degree of steatosis in patients with MAFLD clinically.

## Data Availability Statement

Publicly available datasets were analyzed in this study. This data can be found here: https://www.cdc.gov/nchs/nhanes/index.htm.

## Ethics Statement

The studies involving human participants were reviewed and approved by the NCHS Research Ethics Review Board. The patients/participants provided their written informed consent to participate in this study. Written informed consent was obtained from the individual(s) for the publication of any potentially identifiable images or data included in this article.

## Author Contributions

RJ and XW: study concept and design. XL, JY, and BL: acquisition of data. RJ, XW, LW, FL, and HR: analysis and interpretation of data. RJ, XW, FL, and HR: drafting of the manuscript. FL and HR: critical revision of the manuscript for important intellectual content. All authors have made a significant contribution to this study and have approved the final manuscript.

## Conflict of Interest

The authors declare that the research was conducted in the absence of any commercial or financial relationships that could be construed as a potential conflict of interest.

## Publisher’s Note

All claims expressed in this article are solely those of the authors and do not necessarily represent those of their affiliated organizations, or those of the publisher, the editors and the reviewers. Any product that may be evaluated in this article, or claim that may be made by its manufacturer, is not guaranteed or endorsed by the publisher.

## Footnotes

1
https://www.cdc.gov/nchs/nhanes/index.htm

2
http://www.R-project.org

3
http://www.empowerstats.com

## Abbreviations

MAFLD: metabolic associated fatty liver disease
ASMI: appendicular skeletal muscle index
NHANES: National Health and Nutrition Examination Surveys
NAFLD: Non-alcoholic fatty liver disease
MRI-PDFF: magnetic resonance imaging-derived proton density fat fraction
VCTE: Vibration-controlled transient elastography
NCHS: National Center for Health Statistics
DXA: dual-energy X-ray absorptiometry
BMI: body mass index
BP: blood pressure
TG: triglycerides
HDL-C: high-density lipoprotein cholesterol
FPG: fasting plasma glucose
CRP: C-reactive protein
AI: arm muscle index
LI: leg muscle index
TC: cholesterol
GGT: γ -glutamyl transpeptidase
ALB: albumin
ALP: alkaline phosphatase
UA: uric acid
HbA1c: hemoglobin A1C
BMC: Bone mineral content
OR: odds ratio
FLI: fatty liver index.
